# Dr. G. M. Newton

**Published:** 1859-05

**Authors:** 


DR. G. M. NEWTON, late Emeritus Professor of Anatomy in the Medical College of Georgia, died early in January, at Augusta, of tetanus, occasioned by injuries received when thrown from his buggy. Dr. Newton held the chair of Anatomy in the Medical College of Georgia for nearly twenty years, a post in which he soon gained distinction as an able teacher and elegant lecturer.

